# A dynamic checkpoint in oxidative lesion discrimination by formamidopyrimidine–DNA glycosylase

**DOI:** 10.1093/nar/gkv1092

**Published:** 2015-11-08

**Authors:** Haoquan Li, Anton V. Endutkin, Christina Bergonzo, Arthur J. Campbell, Carlos de los Santos, Arthur Grollman, Dmitry O. Zharkov, Carlos Simmerling

**Affiliations:** 1 Department of Chemistry, Stony Brook University, Stony Brook, NY 11794, USA; 2 SB RAS Institute of Chemical Biology and Fundamental Medicine, 8 Lavrentieva Ave., Novosibirsk 630090, Russia; 3 Novosibirsk State University, 2 Pirogova St., Novosibirsk 630090, Russia; 4 Department of Pharmacological Sciences, Stony Brook University, Stony Brook, NY 11794, USA; 5 Laufer Center for Physical and Quantitative Biology, Stony Brook University, Stony Brook, NY 11794, USA

## Abstract

In contrast to proteins recognizing small-molecule ligands, DNA-dependent enzymes cannot rely solely on interactions in the substrate-binding centre to achieve their exquisite specificity. It is widely believed that substrate recognition by such enzymes involves a series of conformational changes in the enzyme–DNA complex with sequential gates favoring cognate DNA and rejecting nonsubstrates. However, direct evidence for such mechanism is limited to a few systems. We report that discrimination between the oxidative DNA lesion, 8-oxoguanine (oxoG) and its normal counterpart, guanine, by the repair enzyme, formamidopyrimidine-DNA glycosylase (Fpg), likely involves multiple gates. Fpg uses an aromatic wedge to open the Watson–Crick base pair and everts the lesion into its active site. We used molecular dynamics simulations to explore the eversion free energy landscapes of oxoG and G by Fpg, focusing on structural and energetic details of oxoG recognition. The resulting energy profiles, supported by biochemical analysis of site-directed mutants disturbing the interactions along the proposed path, show that Fpg selectively facilitates eversion of oxoG by stabilizing several intermediate states, helping the rapidly sliding enzyme avoid full extrusion of every encountered base for interrogation. Lesion recognition through multiple gating intermediates may be a common theme in DNA repair enzymes.

## INTRODUCTION

**Figure 1.**
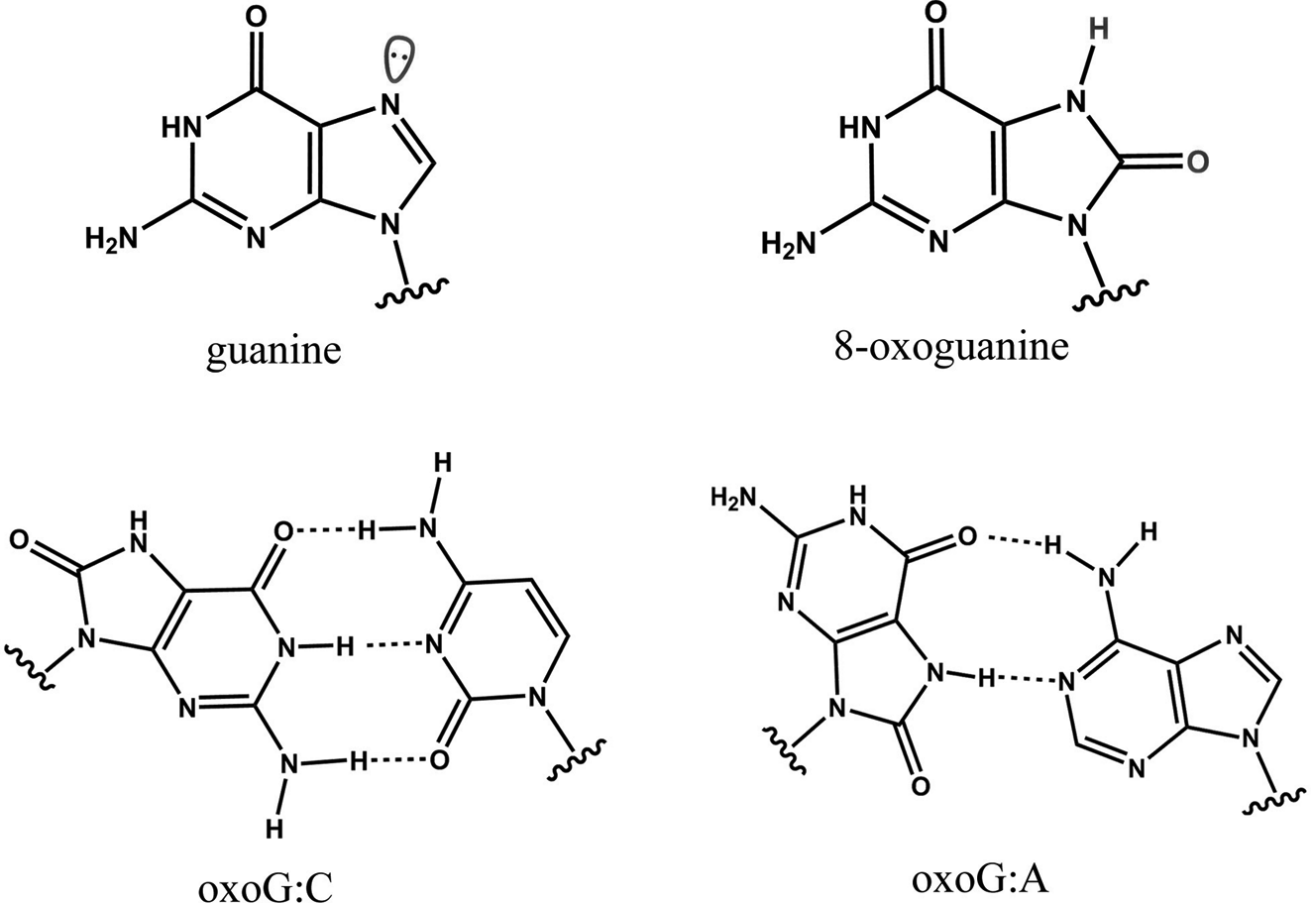
Top: comparison of guanine and oxoG. Bottom: base pairing of oxoG:C and oxoG:A.

Oxidative DNA damage is one of the most common types of damage known to affect the genome (1,2). The main source of oxidative DNA damage is from reactive oxygen species, which arise due to endogenous and exogenous factors, such as aerobic metabolism and exposure to ionizing and photosensitized UV radiation, respectively (3). One common product of reactive oxygen species of particular interest is oxoG, which is formed by oxidation of a normal guanine base at the C8 position, as shown in Figure 1 (4). Eukaryotic cells contain the background level of ∼1 oxoG lesion per 10^6^ guanines, which must be efficiently located and excised to maintain genomic integrity (5). The oxoG lesion remains structurally similar to a normal guanine (G) nucleobase, with a two atom difference on the major groove face in B-form duplex DNA: oxoG has an O^8^ and an H^7^ on N7, whereas G has an H^8^ and a lone pair on N7 (Figure 1). If the oxoG lesion remains uncorrected, it tends to adopt a Hoogsteen orientation and guide incorporation of adenine as a base pairing partner during replication (Figure 1, bottom), promoting a G:C to T:A transversion mutation linked with cancers and various age-related diseases (1,6,7).

Elaborate cellular repair processes have evolved to combat various types of DNA damage with high fidelity, one of which is the base excision repair pathway (BER) (8). In human cells, 11 known BER glycosylases usually process 100 to 10 000 lesions per cell per day (9–11). These glycosylases share similar structural features with their bacterial counterparts, including an intercalating aromatic/aliphatic wedge that enters through the minor groove, an overall bending/kinking of the DNA resulting from binding and a base eversion process that repositions the damaged nucleobase into a protein active site for excision (12,13).

The BER DNA glycosylases that target oxoG as one of their substrates in human and bacterial cells are hOGG1 and Fpg (also known as MutM), respectively. These glycosylases are functional analogs that excise the oxoG lesion when opposite a cytosine in duplex DNA. The catalytic mechanisms of oxoG excision by hOGG1 and Fpg have been well characterized (14–19). Both enzymes carry out nucleophilic attack on the C1′ atom of the deoxyribose sugar of oxoG, initiating base excision, followed by two consecutive steps of β elimination of the 3′ and 5′ phosphate groups (15,16). Although hOGG1 and Fpg have different tertiary structures, they contain analogous residues proposed to perform similar functions during recognition and base eversion. Both enzymes have aromatic intercalating wedges (Tyr203 in hOGG1 and Phe113 in *Geobacillus stearothermophilus* Fpg, respectively) that enter through the minor groove at the site of damage (16,20) and destabilize the interrogated base pair (21). Additionally, arginine side chains (154/204 for hOGG1 and 111 for Fpg) intercalate through the minor groove once the lesion is everted into the active site, recognizing the hydrogen bond acceptor atoms N3 and O^2^ of the cytosine base paired to the oxoG (16,22). The set of hydrogen bonds that form between the arginine residue(s) of each enzyme and the orphan base is specific for a cytosine, and is disrupted by any other nucleobase in this position, thus contributing to a biologically relevant substrate specificity of hOGG1 and Fpg.

Previous crystallographic work has been able to provide putative snapshots along the eversion process (20,23–27). Of particular interest are two crystallographic structures of *G. stearothermophilus* Fpg bound to DNA that define two states: one in which an undamaged G nucleotide is intrahelical (PDB 2F5O) (20), and another where a damaged oxoG is trapped in an extrahelical conformation in the active site of a catalytically impaired mutant enzyme (PDB 1R2Y) (27). Although these two endpoint snapshots are useful for understanding two biologically relevant states, they give an incomplete picture of how Fpg discriminates against G in favor of oxoG during a dynamic process of eversion.

Earlier, we have applied millisecond-resolution stopped-flow kinetics with fluorescence detection to study recognition and removal of damaged bases by several DNA glycosylases, including Fpg (21,28–34). An emerging theme in all DNA glycosylases studied so far is their use of multiple kinetic gates in the reaction: several transient kinetic intermediates are usually detected, each of which can favor productive recognition of good substrates and disfavor nonsubstrate bases. In the case of Fpg, relying on the known structures of artificially stabilized transient states of the recognition pathway (20,23,25–27,35) and using the intrinsic enzyme Trp fluorescence and various fluorescent reporters supplemented by atomistic simulations, it was possible to assign the kinetic intermediates to the known structural intermediates. Notably, two processes that contributed most to the discrimination between good and poor Fpg substrates were insertion of the Phe wedge and eversion of the sampled base. In particular, normal G is not rejected immediately while remaining in an intrahelical conformation but is at least partially everted (21).

We hypothesize that base eversion is needed for oxoG recognition, and at least one or more recognition steps occur at the early stages of base eversion, since the extremely rapid sliding of Fpg along DNA strongly suggests that Fpg does not fully evert every encountered base to the active site (36). However, the eversion process cannot be further decomposed in stopped-flow experiments, since no conformational changes around fluorescent moieties occur between the point when oxoG is unstacked from its neighbors till its entry into the active site. Therefore, in the present work we investigated the eversion pathway for oxoG and G computationally so as to understand when and how it contributes to the substrate specificity of Fpg. Molecular dynamics simulations were used to model the low-populated higher energy states of base eversion whose structures and dynamics are inaccessible by experimental methods. We have modeled the eversion through the major groove, which has been shown to be energetically preferable to the minor groove path (37). The free energy profiles are calculated for eversion from an intrahelical, unopened conformation to an extrahelical conformation, where the target base is bound by the active site loop in its pre-excision complex. The mechanism for oxoG/G discrimination along the eversion pathways can be understood by structural analysis, energy decomposition, as well as biochemical mutation analysis of residues critical for oxoG recognition.

## MATERIALS AND METHODS

The Amber 11 and 12 suites of programs were used for all calculations in this work (38). The ff99SB force field (39) was used with the parmbsc0 backbone modification to the ff99 DNA parameters (40) for all systems. The parameters for oxoG were based on the parameters developed by Miller *et al*. (41). The endpoint structures of the base eversion pathway were generated from two crystal structures of *G. stearothermophilus* Fpg, 2F5O (20) (intrahelical endpoint) and 1R2Y (27) (extrahelical endpoint) in the same way as in our previous work (37). Previously, we set the catalytic Pro1 neutral because we were interested in investigating the scenario directly preceding the catalytic reaction, in which the Pro1 needs to be deprotonated to act as a nucleophile (42). In this work, we focus on the early stages of base eversion process, which are probably prior to the proton rearrangement events at the active site. We used the H++ program, which predicts p*K*_a_ based on the standard continuum solvent methodology and produces results comparable to the experimentally determined values (43). The program estimated a p*K*_a_ of 9.3 for Pro1 in the equilibrated intrahelical structure, and thus here we set Pro1 to be positively charged. The protonation state of other residues, the sequences and initial coordinates of the endpoint structures are the same as in our previous work (37).

The endpoint structures were solvated with TIP3P explicit water (44) in a truncated octahedron with at least 12 Å between solute atoms and the box boundary. Each of the solvated structures was minimized and equilibrated in five stages, with restraint force constant of 100 kcal/(mol·Å^2^) unless otherwise noted: (i) a 10 000-step minimization with positional restraint on the heavy atoms; (ii) a 100 ps MD simulation in which the system was heated linearly to the target temperature of 330 K, which reflects the biological temperature optimum for *G. stearothermophilus*, while the heavy atoms of the complex were restrained; (iii) a 100 ps and a 250 ps MD simulations with the heavy atoms of the complex restrained by 100 and 10 kcal/(mol·Å^2^) restraints, respectively; (iv) a 100 ps, a 200 ps and a 250 ps MD simulations with the heavy atoms of the protein and DNA backbones restrained by 10, 1 and 0.1 kcal/(mol·Å^2^) restraints, respectively; (v) a final 2 ns unrestrained simulation. During minimization and equilibration SHAKE was employed to constrain bonds involving hydrogen atoms (45), and a 1 fs time step was used. The particle mesh Ewald method was used to approximate long-range Columbic interactions (46,47) and the nonbonded cutoff was set to 8 Å. Through step (iii) to (v) constant temperature of 330 K and constant pressure of 1 atm were maintained by the weak-coupling algorithm (48).

The protocol for running NEB (49) path simulations was adopted from our previous work on this system (37), using our partial NEB variant (50) that allows mapping of the pathway in explicit water. The initial temperature was set to 330 K and the spring force constant was 2 kcal/(mol·Å^2^) for the first 100 ps path optimization, then the spring constant was increased to 20 kcal/(mol·Å^2^) for the following 500 ps. The systems were gradually heated to 380 K over the next 100 ps, the temperature was maintained over 200 ps, and then was decreased back to 330 K over the next 100 ps. The final production runs were then performed over 500 ps. During the annealing and the production steps the spring forces were set to 50 kcal/(mol·Å^2^).

**Figure 2.**
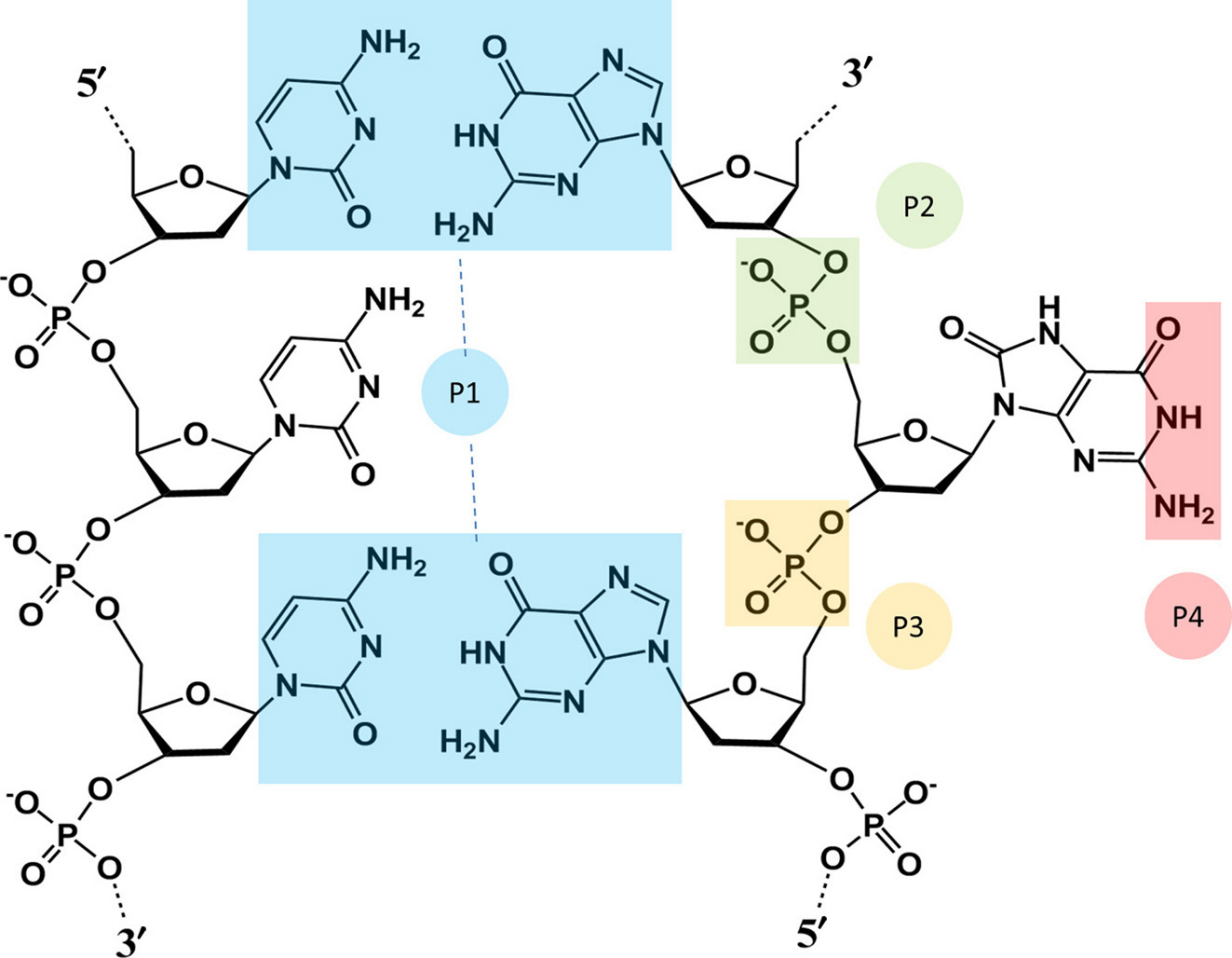
Definition of the eversion angle used for free energy mapping. The four points that define the eversion angle are boxed in different colors: P1, the center of mass (COM) of the four bases flanking the target base pair; P2, the COM of the 5′ phosphate group; P3, the COM of the 3′ phosphate group; P4, the COM of the Watson-Crick edge of the everting base.

We previously developed a modified COM pseudodihedral angle reaction coordinate (CPDb), to describe base eversion (51). CPDb was then used in PMF calculation for oxoG eversion in Fpg (37). In that work, since we were comparing eversion through the minor groove and major groove, we needed to also use a second reaction coordinate for rotation around the glycosidic bond, since the CPDb was not sensitive to such rotation on the minor groove pathway (37). These 2D free energy calculations are computationally demanding as compared to a single reaction coordinate. In this work, by focusing on eversion through the preferred major groove pathway, we were able to develop a modified CPDb (Figure 2, hereafter referred to as the eversion angle), which is more sensitive to the glycosidic rotation of the everted base in the active site (see Supplementary Figure S1 for further details).

Umbrella sampling (US) was used to obtain the PMF as a function of the eversion angle. The US protocol was similar to that in our previous studies of Fpg (37,51). For each WT system, two completely different sets of initial structures were taken from the NEB production trajectories and US was performed independently for 2.5 ns for each set. In each US run, 64 windows were evenly spaced along the eversion angle at ∼4° intervals, and were restrained by 0.183 kcal/(mol·degree^2^) umbrella potential along the eversion distance in an NVT ensemble. The time step was 2 fs and the temperature was maintained at 330 K using a Langevin thermostat (52) with a 75.0 ps^−1^ collision frequency. The eversion angle values were recorded every time step and were analyzed using the weighted histogram analysis method (53). The error bars were then calculated from the difference of the two independent runs. Since the choice of zero point in free energy is arbitrary, the intrahelical endpoint was set to 0 kcal/mol in the PMF to highlight the differences as eversion proceeds.

To explore the structures of the oxoG and the G systems along the base eversion pathways, the US trajectories of these two systems were visualized using VMD (54). Important residue to residue interactions along the base eversion pathways were then identified. To investigate the interactions, distances and nonbonded energies were calculated from the combined US trajectories. The electrostatic and van der Waals interaction terms were independently calculated and combined to give the total nonbonded interaction energy. For each system, the eversion angle and variables (distances and nonbonded energies) were analyzed for each picosecond from their respective US simulations. Each average variable data point was calculated for every 5° bin of eversion angle, and the error bars were calculated from the difference of the two independent US runs. The structures shown were obtained from the US trajectories and are representative structures exhibiting the important interactions. The sequence conservation chart was generated using Weblogo (55,56).

The variants of *Escherichia coli* Fpg harboring mutations of the residues Arg108, Asn168 and Arg258 (corresponding to Arg111, Asn173 and Arg263 of *G. stearothermophilus* Fpg) were produced in pET-28a(+) plasmid using QuikChange Lightning site-directed mutagenesis kit (Agilent Technologies, Santa Clara, CA). Mutant and wild-type Fpg were expressed and purified as described (57). Activity assays were performed on the 5′-^32^P-labeled duplex oligonucleotide substrate with the damaged strand of the sequence 5′-CTCTCCCTTCXCTCCTTTCCTCT-3′ (X = oxoG or AP site) and the opposite strand fully complementary and bearing C opposite to the lesion. To obtain the AP substrate, 10 pmol of the duplex contained uracil in the required position was treated with 1 U *E. coli* uracil–DNA glycosylase (New England Biolabs, Ipswich, MA) immediately before reaction with Fpg. The fraction of the active enzyme in the preparations of wild-type Fpg and all mutants was determined by NaBH_4_ trapping with the AP substrate as described previously (31), and was between 10 and 85% depending on the preparation. In the kinetic experiments, the reaction mixture contained 50 mM Tris-HCl (pH 7.5), 100 mM NaCl, 1 mM ethylenediaminetetraacetate, 1 mM dithiothreitol, 2–100 nM oxoG or AP substrate and 1 nM (for oxoG substrate) or 0.2 nM (for AP substrate) wild-type or mutant Fpg (active form). The reaction was allowed to proceed for 10 min at 30°C and terminated by adding an equal volume of 95% formamide/20 mM EDTA dye and heating at 95°C for 3 min (oxoG substrate) or 1 min (AP substrate). The products were separated by electrophoresis in 20% polyacrylamide gel containing 8 M urea and quantified using Molecular Imager FX system (Bio-Rad, Hercules, CA). The *K*_M_ and *k*_cat_ values were determined from 3 to 5 independent experiments by nonlinear regression using SigmaPlot v9.0 (SPSS, Chicago, IL).

## RESULTS AND DISCUSSION

### Simulation strategy

We hypothesize that lesion recognition may occur at transient points (gates) along the base eversion path, rather than in the fully intrahelical or fully everted endpoints. To understand how Fpg discriminates against G in favor of oxoG, we computationally compared the eversion process of G and oxoG in duplex DNA bound by Fpg. Since base eversion in Fpg occurs on the millisecond timescale (31), and is thus unlikely to be adequately sampled by conventional MD simulations, we used the time-independent NEB and US methods to structurally and energetically characterize the base eversion pathway. These methods were successfully applied to comparing the energetic preference of base eversion through the minor or major groove in Fpg (37). Here, we aim to understand when and how Fpg distinguishes oxoG from G by investigating the structural and energetic differences between the eversion processes for oxoG and G.

### Energy profiles of oxoG and G eversion show differences in free energy

**Figure 3.**
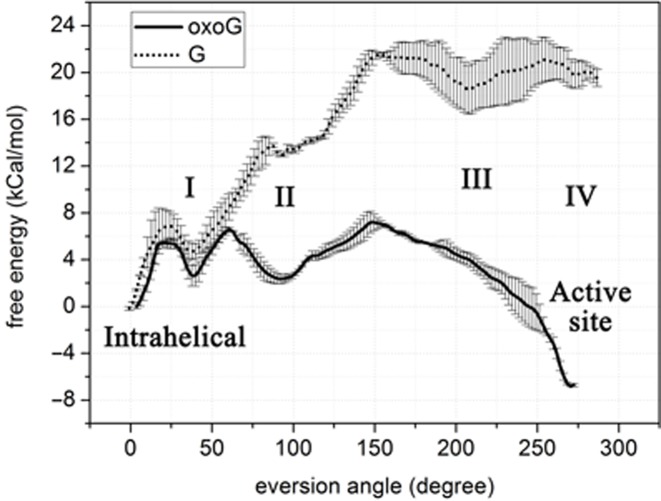
Comparison of free energy profiles for eversion of oxoG (solid) and G (dotted). Four potential oxoG recognition stages are labeled I, II, III and IV. The error bars reflect the precision of the calculated free energies as determined from the difference between two independent runs for each system.

As shown in the free energy profiles (Figure 3), the overall energetic difference between the endpoints of both pathways is consistent with the two crystallographic structures 2F5O (20) and 1R2Y (27), in which G favors the intrahelical state (2F5O) and oxoG, the extrahelical state (1R2Y). Specifically, G favors the intrahelical position by ∼20 kcal/mol with respect to its extrahelical state, whereas for oxoG, the extrahelical position is 7 kcal/mol lower in energy than its intrahelical position (Figure 3). The free energy profiles also suggest that eversion of oxoG is kinetically more favorable than that of G, because the energy barrier to eversion for G (∼22 kcal/mol) is much higher than for oxoG (∼7 kcal/mol). The free energy profiles of the two pathways show significant differences in multiple stages, indicating several potential oxoG-recognizing checkpoints along the base eversion pathway. Examination of these stages may provide insight into the mechanism of lesion recognition. The details of these potential oxoG-recognizing stages are discussed below; for convenience they are referred to as Stage I∼IV (as labeled in Figure 3).

### Discrimination between oxoG and G is present in the early steps of eversion

**Figure 4.**
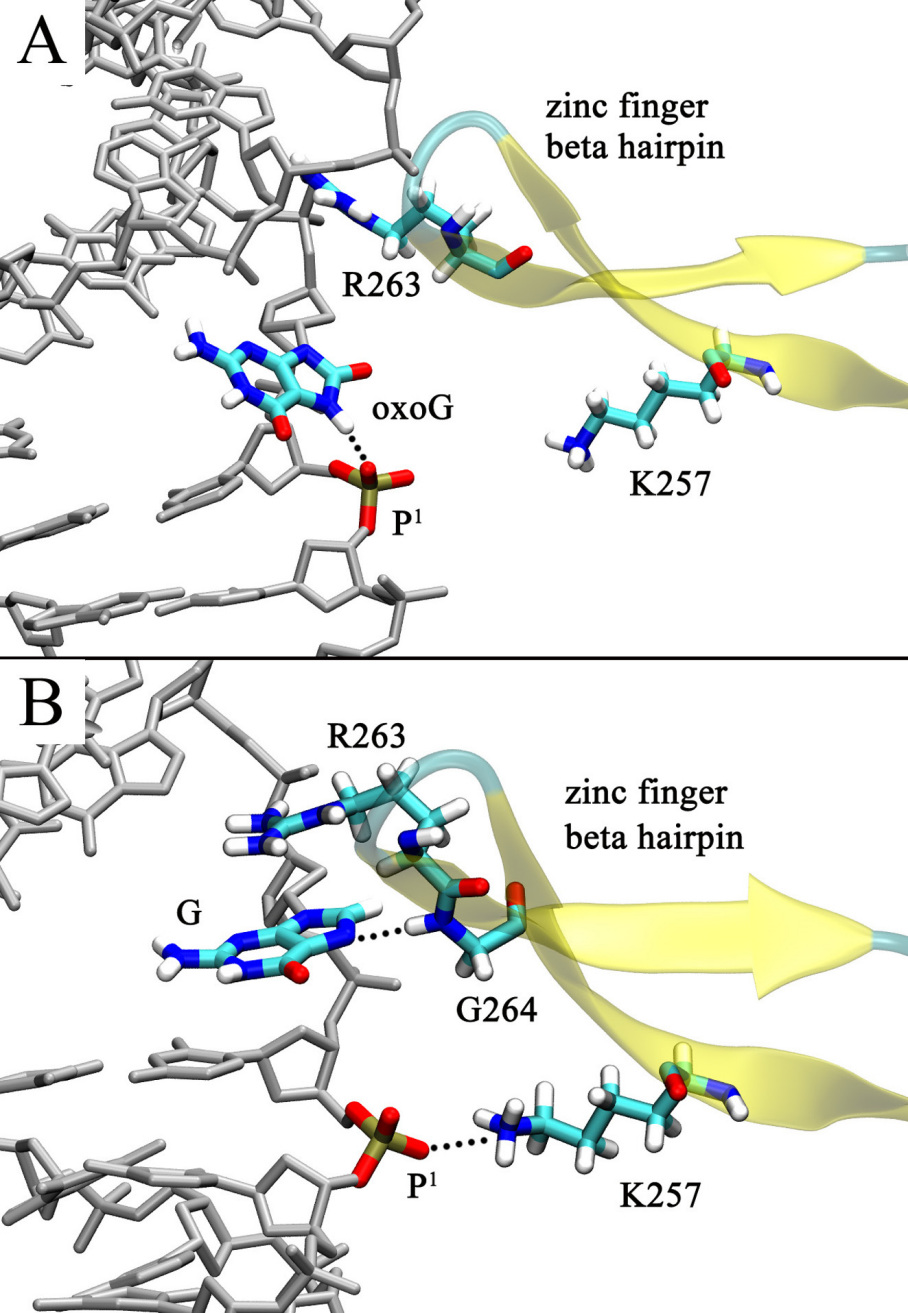
Comparison of the (**A**) oxoG and (**B**) G system structures at the eversion angle of ∼40° (Stage I). Hydrogen bonds are depicted as dotted lines.

After each nucleobase leaves the intrahelical space and enters the major groove eversion pathway, both reach a metastable state of comparable energy (Stage I), indicated by the local energy minimum at an eversion angle of ∼40° (Figure 3, with structures shown in Figure 4). When G reaches the metastable state it forms two significant interactions with the residues on the zinc finger β-hairpin (Figure 4A): first, a cation-π stacking interaction with Arg263, a strictly conserved residue in all Fpg proteins (Supplementary Figure S2), and second, a hydrogen bond between the N7 atom on the imidazole ring of G and the backbone amide H of Gly264. However, N7 of oxoG is protonated, precluding the formation of a comparable hydrogen bond to Gly264. To more fully characterize the oxoG/G-Gly264 interaction along the base eversion pathway, we measured the average distances between the backbone N atom of Gly264 and the N7 of oxoG/G during each US window; this distance is indeed significantly larger for oxoG than for G (Supplementary Figure S3A). Furthermore, the cation-π stacking between Arg263 and G is also absent with oxoG, as suggested by the lower nonbonded energy of Arg263-oxoG compared to that of Arg263-G (Supplementary Figure S3B). This is likely because oxoG moves away from Arg263 to form a hydrogen bond between the protonated N7 of oxoG and the second 5′ phosphate (hereafter referred to as p^1^, Figure 4B). This N7-p^1^ hydrogen bond is not possible with G since the N7 of G is deprotonated. Supplementary Figure S4 demonstrates that the N7-p^1^ hydrogen bond in oxoG is formed at the eversion angle of ∼40°, whereas in the G system the N7-p^1^ distance is much larger in this region. It is possible that the repulsive N7-p^1^ interaction in the G system pushes G toward Arg263 and Gly264. Overall, in this initial stage of eversion, each base forms interactions that specifically probe the protonation status of N7, while maintaining a similar energy to each other and to their intrahelical states, suggesting similar, rapid base pair opening propensity by Fpg.

**Figure 5.**
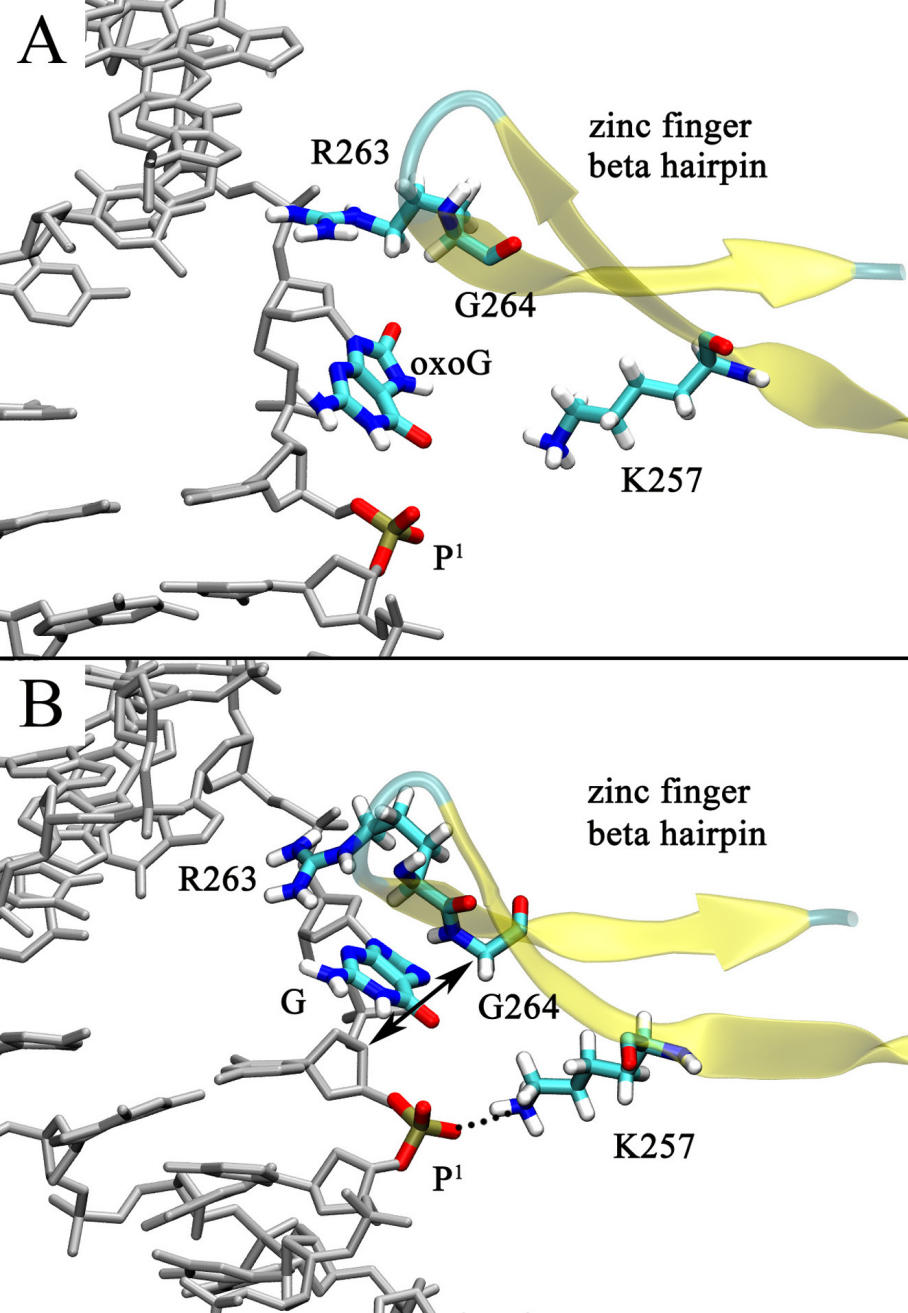
Comparison of the (**A**) oxoG and (**B**) G system structures at an eversion angle of ∼75° (Stage II). The distance between the C3′ of the 5′ nucleotide of the oxoG/G and the Cα of Gly264 (denoted by double arrow) are used to estimate the gap between the zinc finger hairpin and the DNA.

After stage I, further progress in eversion requires that the base pass through the gap between the DNA backbone and the zinc finger hairpin (Figure 5), corresponding to an eversion angle range of 60∼90°. The PMF of G at this transition region shows a significant difference from that of oxoG (Figure 3); G has to overcome a 10 kcal/mol energy barrier (compared to ∼4 kcal/mol for oxoG), suggesting the progressing past stage I is much less favorable for G than for oxoG. Two main factors may contribute to this kinetic discrimination. First, there is a repulsive interaction between p^1^ and the O6/N7 atoms of G, as suggested by their unfavorable pair-wise nonbonded energy in this region of the PMF (Supplementary Figure S5A). In contrast, oxoG does not have as strong repulsive electrostatic interaction with p^1^ since there is no lone pair on N7. Moreover, proximity of p^1^ to G is fixed by the close contact of Lys257 (Figure 5A); whereas with oxoG, p^1^ is disengaged from Lys257 (Figure 5B) and thus p^1^ is has more freedom to move away from O^6^ to avoid unfavorable interactions. As a result, the distance between O6 of oxoG/G and Lys257 is larger in the oxoG system than in the G system (Supplementary Figure S5B).

A second factor in selective hindering of G eversion arises from sterics, since the gap between DNA and the zinc finger hairpin is wider in the oxoG than the G system. In the G system, the hairpin is oriented close to the DNA backbone, whereas in the oxoG system, Arg263 and Gly264 do not closely interact with oxoG, thus the hairpin can move further away from the DNA (Figure 5A and B). To roughly quantify this difference, we calculated the distance between C3′ of the 5′ nucleotide of the oxoG/G and the Cα of Gly264 (denoted by arrows in Figure 5A), which indicates that the gap in the oxoG system is significantly larger than in the G system throughout the intrahelical endpoint to Stage II (Supplementary Figure S6).

We suggest two reasons for the existence of the larger gap in the oxoG system. First, in the intrahelical endpoint, the 5′-side of oxoG is untwisted more than that of G. As shown in Supplementary Figure S7, the twist angle between the target and the 5′ base is significantly smaller in the oxoG system than in the G system. Thus, the DNA strand containing oxoG rotates away from the zinc finger hairpin, making a wider gap than in the G system. This finding is consistent with a recent computational study indicating that oxoG in duplex DNA induces untwisting to its 5′-side base step because of the base−sugar (O^8^ of oxoG and O4′ of the sugar ring) repulsion (58). Second, as discussed above, in Stage I, G closely interacts with Arg263 and Gly264, which may draw the zinc finger hairpin nearer to DNA. Therefore, the probing of protonation of N7 in Stage I may modulate the width of the base passage gap, and thus also influence the steric filtering in Stage II (Figure 5).

**Figure 6.**
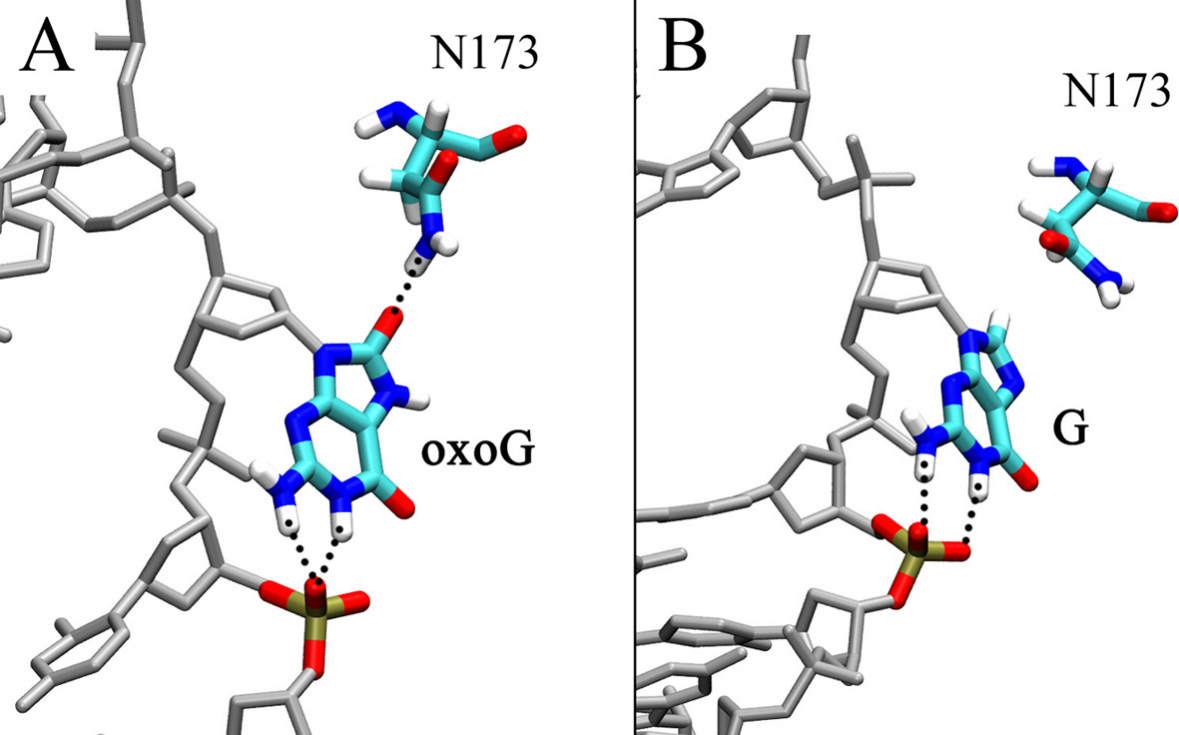
Comparison of the (**A**) oxoG system and the (**B**) G system structures at the eversion of ∼90° (Stage II). Asn173 forms a hydrogen bond that is specific to oxoG.

The eversion PMF for oxoG shows a significant local energy minimum at Stage II (eversion angle of ∼90°, Figure 3), probably because oxoG is stabilized by interactions with p^1^ and Asn173. The Watson-Crick edge of oxoG forms two hydrogen bonds to p^1^ and the O^8^ of oxoG hydrogen bonds to the side chain amide of Asn173 (Figure 6A). Moreover, Asn173 has favorable interaction with oxoG during early stages of eversion before Stage II (Supplementary Figure S8), and this interaction becomes strongest when the O^8^-Asn173 hydrogen bond is present. However, while G also forms two hydrogen bonds to p^1^ at an eversion angle of ∼90°; G does not hydrogen bond to Asn173 since G lacks O^8^ (Figure 6B). As a result, Asn173 directly probes the chemical differences in G/oxoG and contributes to the free energy difference between oxoG and G at Stage II. The calculations therefore suggest that Asn173 is an important component of the oxoG/G discrimination mechanism in Stage II.

Recently published work suggested a minor groove eversion pathway in Fpg, identifying a partially everted G in the minor groove pathway as a putative intermediate (25,26). The enzyme in this structure was covalently bound to the DNA using disulfide cross-linking technology where a N173C mutation was made and a crosslink was formed between residue 173 and the DNA backbone (25). This work assumed that Asn173 does not play a significant role in base eversion, nor did it take into account that an N173C cross-link may sterically block the major groove pathway. Since we identify Asn173 as a key residue in recognition via a major groove path, the minor groove intermediate obtained by mutation of this residue may not reflect a biologically relevant pathway.

### Differences in the free energy profiles late in the eversion pathway

**Figure 7.**
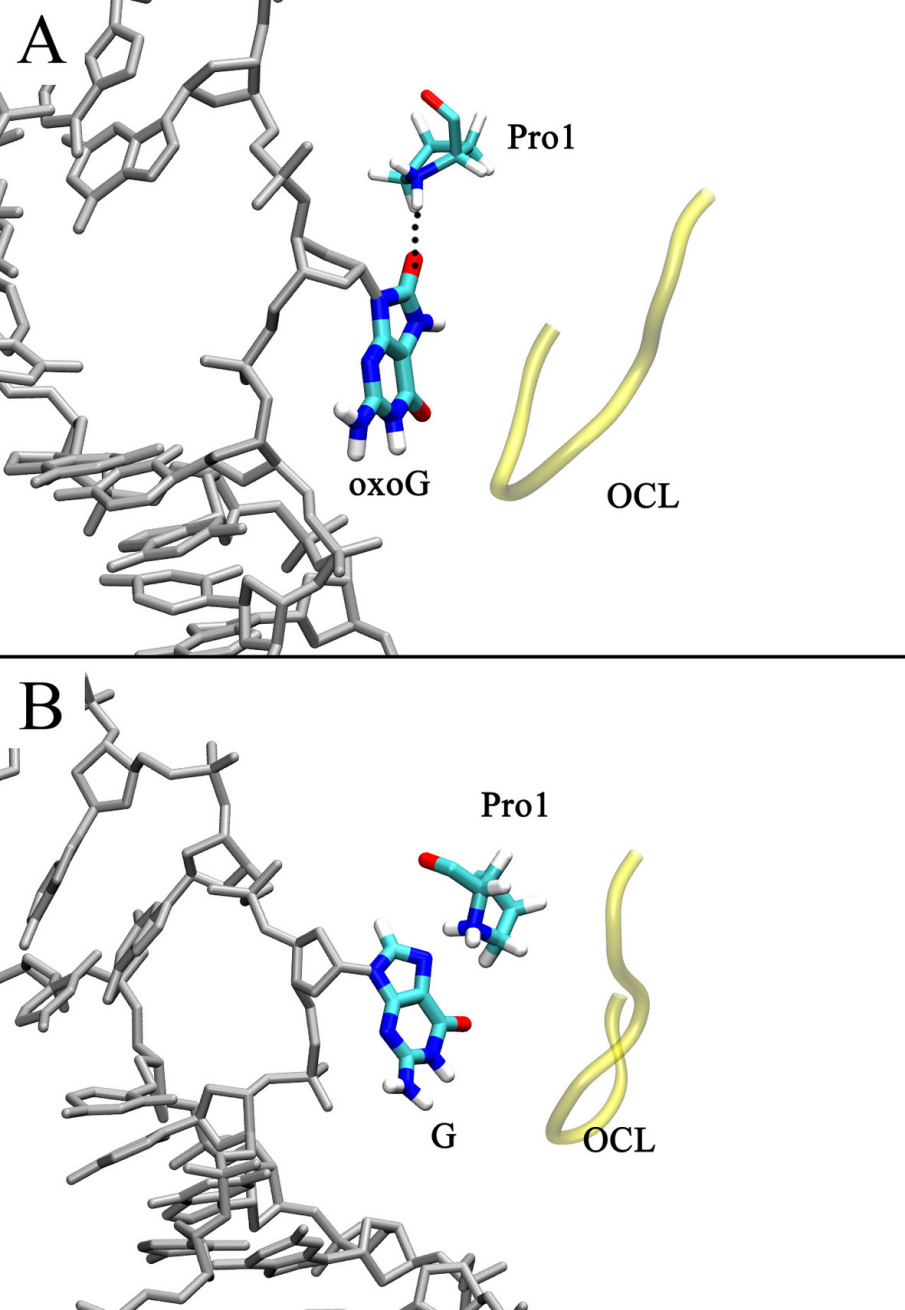
Comparison of the (**A**) oxoG system structure at the eversion angle of ∼220° and the (**B**) G system structure at the eversion angle of ∼200° (B).

To pass Stage II further toward the active site, G has to overcome another barrier of ∼9 kcal/mol, after which the free energy remains roughly stable (Figure 3); whereas oxoG only needs to overcome an energy barrier of 5–6 kcal/mol, and then the free energy oxoG drops by ∼14 kcal/mol from the eversion angle of ∼150° to ∼280° (from Stage III to IV, Figure 3). The energy drop in the oxoG system is likely driven by stabilization of the O^8^ in oxoG by the positively charged Pro1 when transiting to the active site (Figure 7A). Calculation of pairwise nonbonded energy between oxoG and Pro1 supports this hypothesis, showing that the favorable oxoG/Pro1 interaction emerges early in base eversion and becomes stronger as the base flips; as expected, the interaction is strongest when O^8^ of oxoG hydrogen bonds to Pro1 at the eversion angles of 220°–280° (Supplementary Figure S9A). Pro1 also makes a hydrogen bond to the N7 of G (Figure 7B), contributing to the energy minimum at the eversion angle of 200° in the PMF (Figure 3). However, compared to oxoG, the interaction of Pro1 with G is less favorable, especially after the eversion angle of 100°, as indicated by the nonbonded energy (Supplementary Figure S9A). Although the partial charges of the two atoms are similar in the force field (1.013 and 1.043 for O^8^ and N7, respectively), the difference likely arises because O^8^ is an exocyclic atom, whereas N7 is a ring atom, and thus Pro1 can approach closer to O^8^ than to N7 during base eversion. Indeed, in most areas of the eversion path, the O^8^-Pro1 interaction in the oxoG system has closer distance than in the N7-Pro1 in the G system (Supplementary Figure S9B). This difference in the interaction with Pro1 likely contributes to the divergence of the free energy of oxoG and G in Stage III.

**Figure 8.**
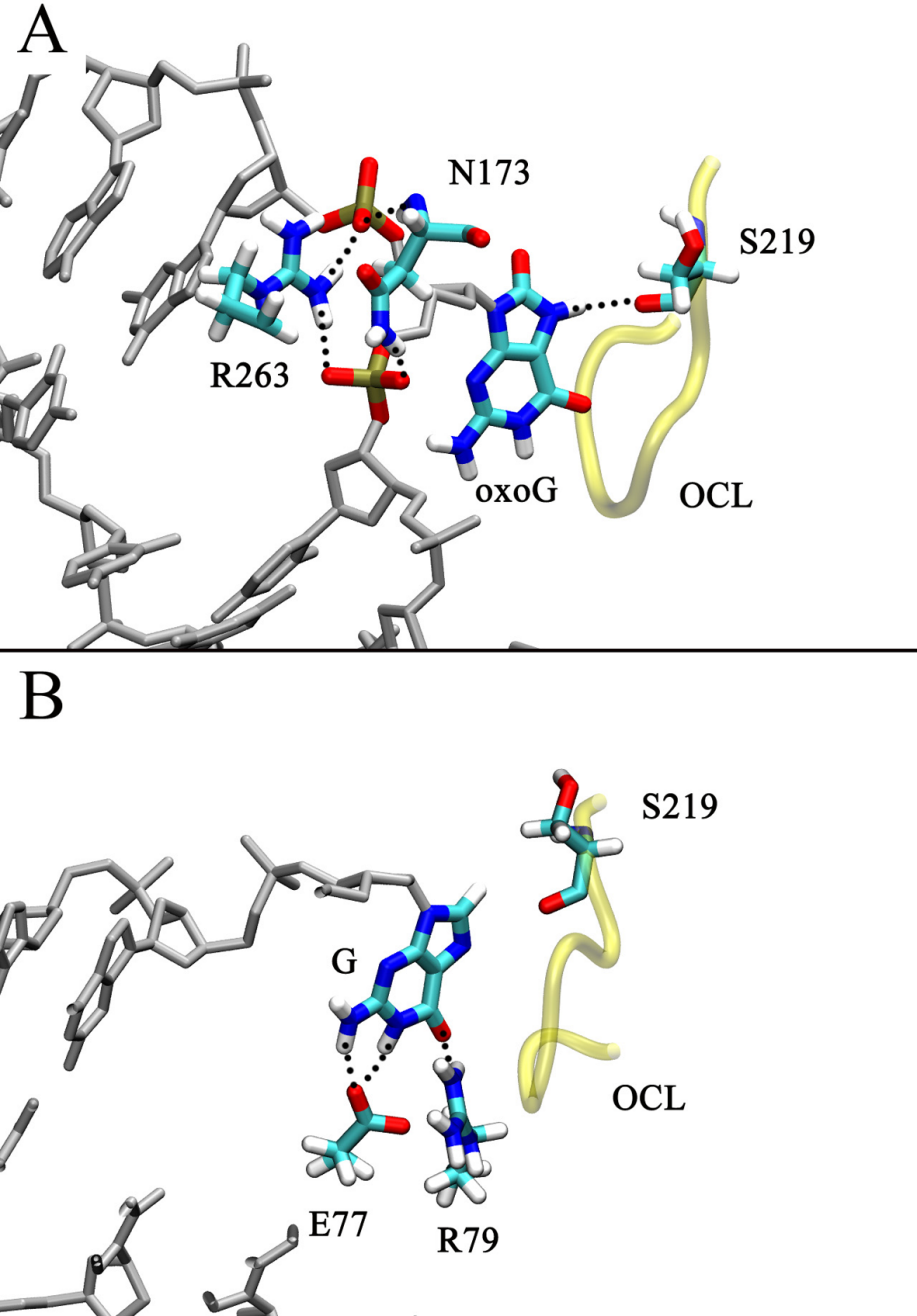
Comparison of the (**A**) oxoG system structure at the eversion angle of ∼275° and the (**B**) G system structure at the eversion angle of ∼280° (B). For clarity, only the OCL and part of the DNA are shown.

The base eversion pathway ends in the enzyme's active site (at the eversion angle of ∼260–280°, Stage IV). As indicated by the free energy profile, Stage IV is a stable state for oxoG. In our simulations, the N7 of oxoG hydrogen bonds to the backbone amide of Ser219, and the O^6^ of oxoG is contacted by the backbone amides of the oxoG-capping loop (OCL). These oxoG-stabilizing interactions reproduce those seen in the crystal structure 1R2Y (27), in which oxoG is interrogating in the active site of E2Q Fpg mutant; thus our results support that the E2Q Fpg-oxoG complex is a good model of the WT system. In addition, the backbone amide and the side chain of Asn173 hydrogen bond to the 3′- and 5′ phosphate group (p^−1^ and p^0^), respectively; and Arg263, stacking with Asn173, also contacts p^−1^ and p^0^ (Figure 8A). Thus Asn173 and Arg263 help to stabilize the everted nucleotide. Surprisingly, when G enters the active site, it does not form any hydrogen bonds to OCL (Figure 8B), probably because the deprotonated N7 of G is repelled by the carbonyl of Ser219 (Supplementary Figure S10), and G rotates to make contacts with Arg79 and Glu77 with its Watson-Crick edge (Figure 8B). Nevertheless, it would be a very rare occasion that a G could overcome the ∼20 kcal/mol energy barrier to reach the active site, therefore the contacts made to G in this state are likely not as important for lesion recognition as the filtering that occurs during the earlier stages of base eversion.

### Arg111 interacts with the cytosine opposite oxoG and may promote oxoG eversion

**Figure 9.**
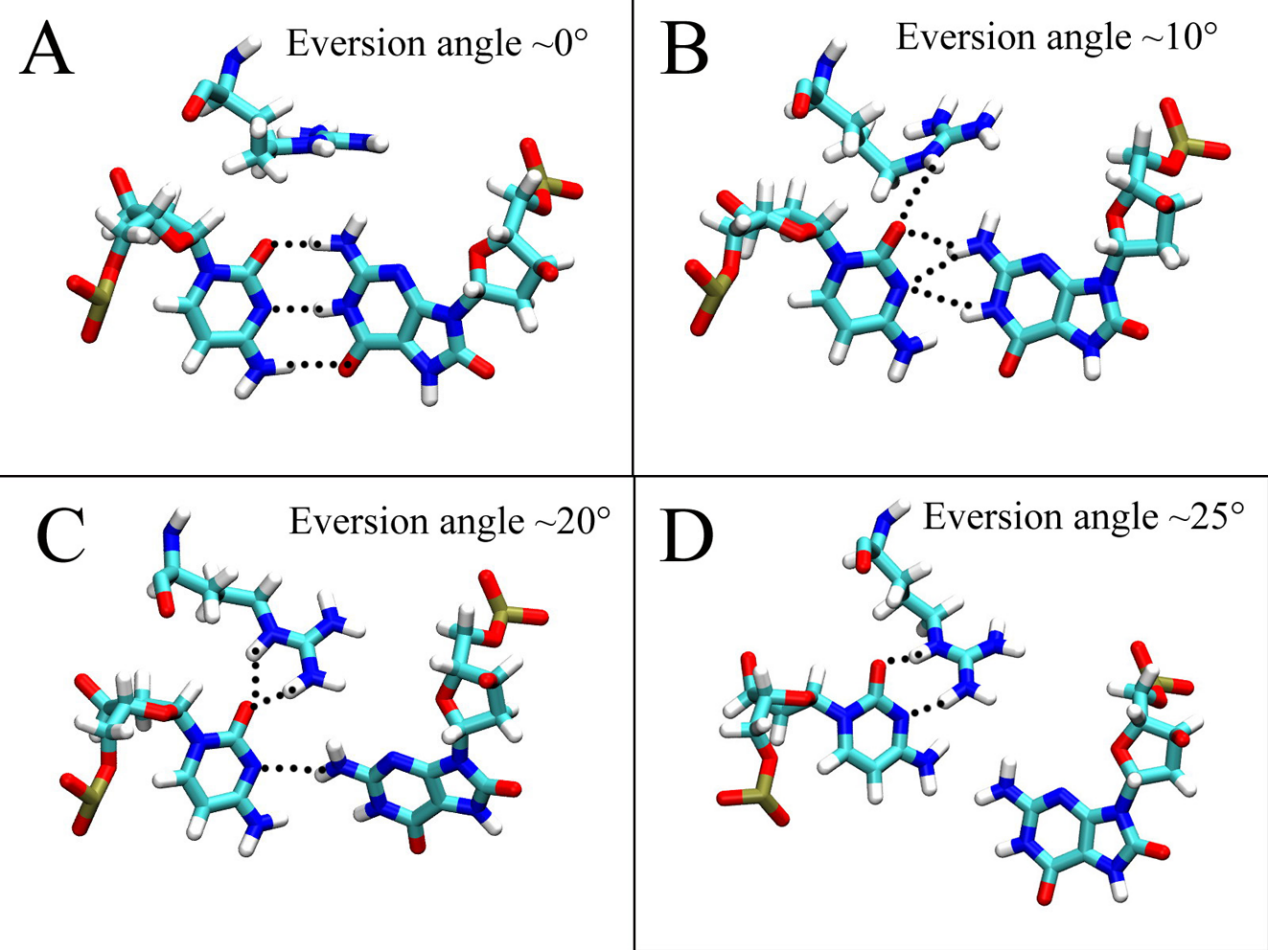
Structures of R111 contacting both the orphan C and the phosphate of the everted oxoG during the early stages of base eversion.

Recognition of the C opposite oxoG is also important for damage repair by Fpg, since removal of an oxoG opposite a base other than C (such as A) can promote mutation. Crystallographic studies have indicated that Arg111 recognizes the C opposite oxoG by bidentate hydrogen bonds with the Watson-Crick edge of the C (27). Previous simulations sampling oxoG eversion through the minor groove showed Arg111 first contacting the O^2^ atom of the C, competing with the intrahelical oxoG and then invading the DNA and forming bidentate hydrogen bonds, concomitant with the base pair opening (26). Nevertheless, the role of Arg111 in the major groove eversion path, which is energetically more favorable than the minor groove one, (37) was unclear. Here our simulations indicate that Arg111 in the major groove path plays a similar role as shown for the minor groove path – it recognizes the opposite C in early stages of base eversion and may promote oxoG eversion by competing with oxoG for hydrogen bond interactions with the C (Figure 9). First, Arg111 is occluded from the DNA when the oxoG:C is intact (Figure 9A). When oxoG slightly shifts away from the original position, Arg111 moves close to O^2^ of the C and starts competing with N^2^ of the oxoG (Figure 9B). As the oxoG flips further, Arg111 comes closer to C and forms two hydrogen bonds to O^2^ (Figure 9C). The bidentate hydrogen bonds between Arg111 and the C are then developed further as the oxoG is disengaged from the paired C (Figure 9D). Thus, it appears that Arg111 competes with oxoG for hydrogen bonding to the opposite C and probably aids the wedge in its role of disrupting the interrogated base pair (21), promoting oxoG eversion.

### Experimental validation of the computational predictions using Fpg mutants

Among the key residues in the proposed lesion discrimination stages, Arg111, Arg263, Asn173 and Pro1 are strictly conserved among Fpg (Supplementary Figure S2), supporting the prediction of their important roles in Fpg function. In order to experimentally verify the predicted criticality of these residues in lesion recognition, we produced a set of corresponding mutations in Fpg from *E. coli*, an enzyme extensively characterized biochemically. Arg108 (Arg111 in *G. stearothermophilus* Fpg) was mutated into Lys (to conserve steric bulk, polarity and charge), Gln (to conserve bulk and polarity, but not charge) or Leu (to conserve bulk only). Asn168 (Asn173 in *G. stearothermophilus* Fpg) was mutated into Asp to eliminate hydrogen-bonding donor capacity and introduce charge, or Gln to increase bulk moderately. Finally, Arg258 (Arg263 in *G. stearothermophilus* Fpg) was mutated into Lys, Gln or totally eliminated by conversion into Ala. Mutation studies of Pro1 was not performed, because Pro1 is required for catalytic activity. The proteins were purified and their steady-state kinetics were determined.

**Figure 10.**
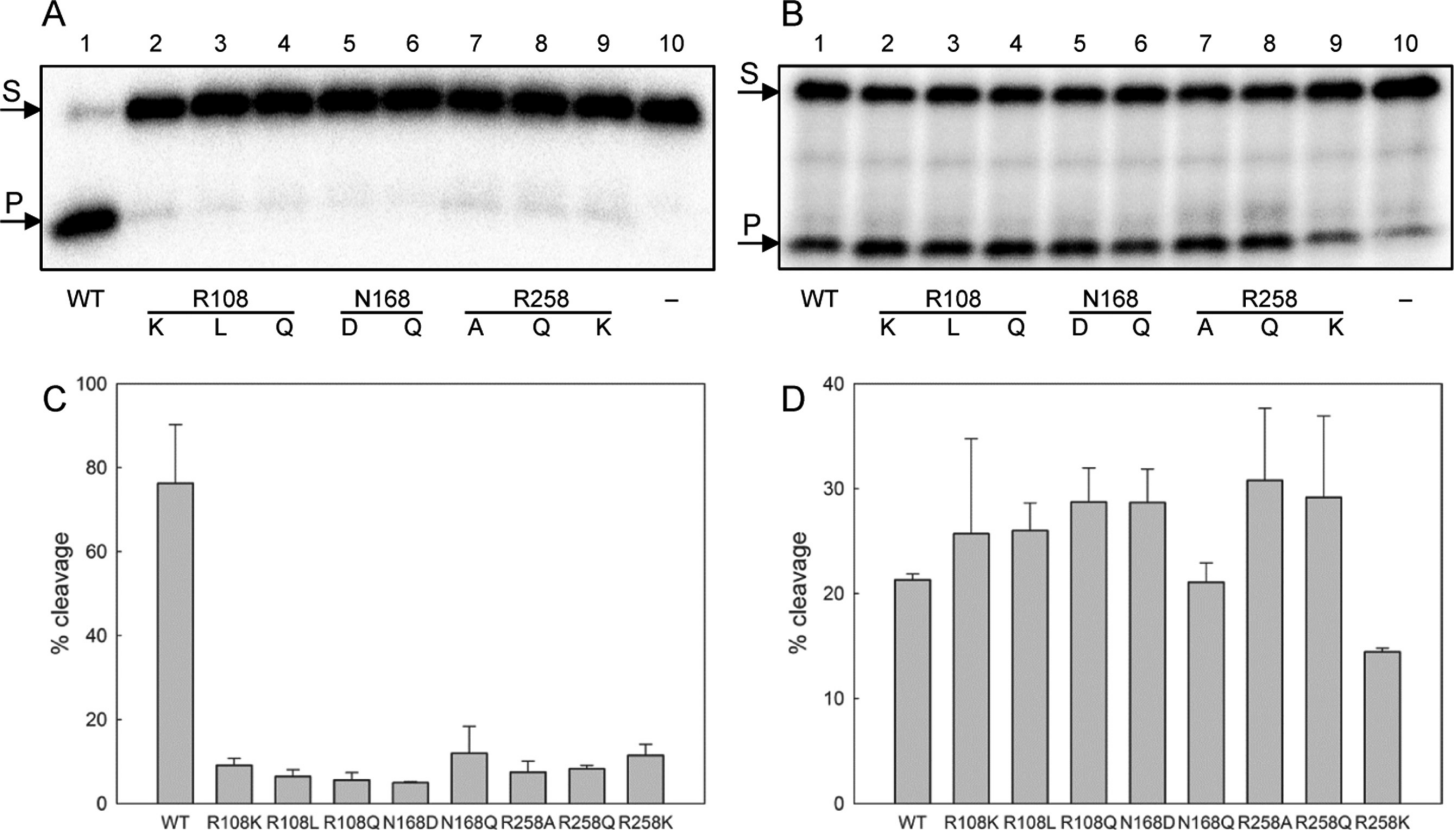
Cleavage of oxoG substrate (**A** and **C**) and AP substrate (**B** and **D**) by wild-type and mutant Fpg proteins. Representative gels (A,B) and mean ± SD (n = 3) are shown. The concentration of the substrate was 50 nM in all experiments, Fpg was taken at 2 nM (oxoG substrate) or 0.2 nM (AP substrate). S, substrate, P, cleaved product.

All mutants were clearly compromised in their ability to cleave oxoG-containing DNA (Figure 10A and C). Characteristically, the activity on the AP substrates was affected to a much lesser degree, if at all (Figure 10B and D). This conservation strongly suggests that the mutations do not disturb the general enzyme structure, its ability to bind DNA or position the catalytic residues in a proper conformation and that their detrimental effect is likely due to the disruption of the base eversion process. AP sites are known to exist in a spontaneous dynamic equilibrium between intra- and extrahelical states, (21,59) and thus the requirements for active enzyme-induced eversion are likely relaxed. The mutants could be also cross-linked to the AP substrate by NaBH_4_ treatment (not shown), providing a way to quantify the fraction of the active enzyme.

The kinetic constants obtained for oxoG substrate after normalization for the active enzyme concentration support the role of Arg108, Asn168 and Arg258 in the eversion process (Table 1). The mutations hardly affected *K*_M_, in the cases when it could be measured independently, again indicating that the overall affinity for the substrate and stability of the enzyme–substrate complex did not change much. On the contrary, the catalytic constant was down at least 10-fold, suggesting the disruption of the events following substrate binding. The least affected was the N168Q mutant, which still has the ability to form all hydrogen bonds but presumably does it in a less favorable conformation due to the extra methylene group. On the other hand, the N168D mutation, abolishing the transient hydrogen bonds with O^8^ and DNA phosphates and introducing an unfavorable electrostatic repulsion of oxoG, was the most detrimental, matching the predictions of simulations. Of all Arg258 mutants, the R258Q retained most activity, again in agreement with the modeling results, which show importance of pi stacking and hydrogen bonding with this residue. Electrostatic interactions alone do not seem to play a significant role in the oxoG guidance through the eversion pathway, since the substitution of Lys for Arg258 had the same effect as the abolition of Arg258 altogether. Finally, Arg108 mutants retained minor residual activity only if the charge was conserved. As shown in Figure 9, the equivalent arginine, Arg111, first anchors itself to the lone pair of O^2^ of the C opposite to the lesion and acts as a lever in penetrating the base stack and then competes with oxoG for the Watson–Crick bonding edge of the opposite C. Obviously, Lys could also form a bond to O^2^ and penetrate the helix, partially destabilizing the intrahelical oxoG:C base pair, but fails to form the C-specific bonds later.

**Table 1.** Wild-type and mutant *Escherichia coli* Fpg kinetic parameters

Enzyme	*K*_M_, nM	*k*_cat_, s^−1^	*k*_sp_, nM^−1^·s^−1^	Relative activity, %
Wild-type	8.1 ± 2.3	1.7 ± 0.1	0.21 ± 0.06^a^	100^c^
R108K	18.0 ± 4.0	0.070 ± 0.006	0.0039 ± 0.0009	1.9
R108L	n/d^b^	n/d	(6.4 ± 0.4) × 10^−4^	0.3
R108Q	n/d	n/d	(6.4 ± 0.3) × 10^−4^	0.3
N168D	n/d	n/d	(4.7 ± 0.2) × 10^−4^	0.2
N168Q	8.1 ± 1.9	0.16 ± 0.01	0.020 ± 0.005	9.5
R258A	n/d	n/d	(7.6 ± 0.3) × 10^−4^	0.4
R258Q	6.3 ± 3.0	0.08 ± 0.01	0.013 ± 0.006	6.2
R258K	n/d	n/d	(6.0 ± 0.4) × 10^−4^	0.3

^a^*k*_sp_ = *k*_cat_/*K*_M_.

^b^Cleavage too low to separately calculate *K*_M_ and *k*_cat_; *k*_sp_ calculated from the slope of the linear part of the *v*_0_ versus [S] dependence.

^c^Calculated from the ratio of the respective *k*_sp_ values.

### A proposed oxoG/G discrimination mechanism of Fpg

Based on the findings discussed above, here we propose an oxoG/G discrimination mechanism for Fpg. In the intrahelical state, oxoG induces untwisting to the 5′ base step, pushing the DNA backbone further away from the zinc finger hairpin as compared to that of the G system. Arg111 approaches the C opposite the oxoG and helps the wedge to disrupt the base pair. As oxoG opens from the pair, it quickly enters a metastable state in which it is stabilized by the DNA backbone phosphate p^1^, whereas G is also in a metastable state with stabilizing interactions from Arg263 and Gly264, which may help to draw the zinc finger hairpin nearer to DNA as compared to that of the oxoG system, and thus trapping G by sterically hindering G from further eversion (Stage I). The wider hairpin-DNA gap in the oxoG system allows oxoG to pass through with significantly lower energy barrier as compared to G. OxoG then enters the second metastable state where it is stabilized by p^1^ as well as a hydrogen bond between O^8^ and Asn173, which is not observed in the G system (Stage II). Further eversion of both bases is then facilitated by the interaction from Pro1, which is stronger in the oxoG system than in the G system (Stages IV). Ultimately, oxoG enters the active site and is contacted by Ser219 and other interactions from the oxoG-capping loop, whereas G is rejected by the OCL, probably due to the unfavorable interaction from Ser219, and instead stays in an adjacent position contacted by Arg79 and Glu77 (Stage IV).

A similar mechanism with multiple gates along the eversion pathway may be universal for other glycosylases, as pointed out by structural and computational evidence. Probably the best-characterized example so far is hOGG1, which possesses an ‘*exo*-site,’ which traps adventitiously everted G in the lowest energy state but is only fleetingly visited by everted oxoG (60,61). A later gate with oxoG near its final position in the active site but still lacking the complete set of base–protein bonds was also observed structurally (62). Finally, in the active site G is both energetically destabilized and misaligned (61,63). A less detailed eversion pathway has been established for human uracil–DNA glycosylase (hUNG), where an *exo*-site has also been identified in a crystal structure (64), although the energetic preference of normal and damaged pyrimidines for this site has not been established. The active site of hUNG is structurally optimized for binding the uracil base, with sterical clashes and repulsive interactions disfavoring binding of C and T (65). Solving the structures of other nucleotide-flipping enzymes (other DNA glycosylases, AP endonucleases, DNA methyltransferases, DNA demethylases, DNA deaminases) with target and non-target DNA at various snapshots along the reaction pathway is technically challenging but possible, given the successful examples of Fpg, hOGG1 and hUNG, and will produce the definitive answer about the generality of the multigate eversion mechanism.

The concept of dynamic enzyme–DNA recognition in its present form, an extension of the venerable induced-fit theory, was shaped in 1990s when structures of many enzyme–DNA complexes were determined and shown to contain highly distorted DNA and sometimes protein, which clearly could not be achieved in a single concerted step (66). In addition to DNA glycosylases, AP endonucleases, DNA methyltransferases, DNA demethylases and DNA deaminases, for which target nucleotide eversion have been demonstrated, main aspects of dynamic recognition are considered for systems without eversion but still highly distorting, such as DNA polymerases (67), nucleotide excision repair factor UvrA_2_B (68), transcription factors (69), restriction endonucleases (70), etc. It has long been held that, since the rate of an enzymatic reaction ultimately depends on the overall barrier height between enzyme–substrate and enzyme–product ground states, transient intermediates have no effect on substrate discrimination. However, an emerging picture of enzyme–substrate complexes populating a wide rugged landscape of conformational states (71–73) suggests that, rather than enhancing the reaction rate for the substrate, the transient states may be required for kinetic trapping of numerous nonsubstrates to divert them from the path leading to the productive complex. This is especially important for DNA repair systems where the error cost is very high (74). The checkpoints along the nucleotide eversion path are thus important safeguards that may critically contribute to the precision of action of the enzymes that maintain our genomes.

## CONCLUSION

To understand how Fpg efficiently recognizes oxoG from a vast excess of undamaged guanines, we modeled the free energy pathway of eversion for both the oxoG lesion and an undamaged G using MD simulations. In addition to free energy profiles, structural analysis of the simulation data revealed specific interactions recognizing oxoG. Arg111 recognizes the C opposite the oxoG and probably promotes opening of the oxoG:C pair. Early interaction with the second 5′ phosphate specifically recognizes the protonated N7 of oxoG; Asn173 and Pro1 play a role in recognizing O^8^ of oxoG and thus facilitating further eversion of oxoG. We then used biochemical mutation analysis of residues to verify the critical roles of Arg111, Asn173 and R263 in oxoG eversion. On the other hand, eversion of the undamaged G is hindered by the unfavorable early interactions from the DNA backbone, and the narrow gap between DNA and the zinc finger beta hairpin also prevents G from proceeding along the eversion pathway. Therefore, Fpg can discriminate against G in favor of oxoG in early stages of base eversion, and this early lesion discrimination process is much more efficient than the one occurring in the active site, allowing Fpg to quickly detect oxoG during fast sliding.

## Supplementary Material

SUPPLEMENTARY DATA
