# Supplementary material for: A dynamic checkpoint in oxidative lesion discrimination by formamidopyrimidine–DNA glycosylase
Source: Nucleic Acids Res. 2015 Nov 8;44(2):683–94. doi: 10.1093/nar/gkv1092 (PMC4737139; doi:10.1093/nar/gkv1092)
Supplement: SUPPLEMENTARY DATA [file supp_44_2_683__index.html]

A dynamic checkpoint in oxidative lesion discrimination by formamidopyrimidine–DNA glycosylase — SUPPLEMENTARY DATA 

# A dynamic checkpoint in oxidative lesion discrimination by formamidopyrimidine–DNA glycosylase

## SUPPLEMENTARY DATA

- SUPPLEMENTARY DATA
