# Supplementary material for: A dynamic checkpoint in oxidative lesion discrimination by formamidopyrimidine–DNA glycosylase
Source: Nucleic Acids Res. 2015 Nov 8;44(2):683–94. doi: 10.1093/nar/gkv1092 (PMC4737139; doi:10.1093/nar/gkv1092)
Supplement: SUPPLEMENTARY DATA [file supp_gkv1092_nar-02643-f-2015-File012.pdf]

**Supporting Information for**

# A dynamic checkpoint in oxidative lesion discrimination by the formamidopyrimidine–DNA glycosylase

Haoquan Li, Anton V. Endutkin, Christina Bergonzo, Arthur J. Campbell, Carlos de los Santos, Arthur Grollman, Dmitry O. Zharkov, Carlos Simmerling

The comparison of our previously used CPDb with the newly developed eversion angle in describing the path of oxoG eversion is shown in **Figure S1**. In the region near the extrahelical endpoint (right side of graph), CPDb remains ~270° while the glycosidic angle changes from ~0° to ~70°, indicating that CPDb cannot adequately describe changes during the final 70° rotation of the glycosidic bond, representing the process of the base entering the active site,. On the other hand, the glycosidic rotation from ~0° to ~30° near the extrahelical endpoint remains correlated with continued change of the eversion angle from ~250° to ~270°, which is an improvement over CPDb. The eversion angle is not as sensitive to the final glycosidic rotation from ~30° to ~70°, but these changes are likely normal fluctuations of the base in the active site, supported by our previous observation that the free energy landscape is relatively flat from ~30° through ~70° rotation of the glycosidic bond of oxoG in the Fpg active site.[1] Although the eversion angle is not sensitive to these base fluctuations in the active site, it can still describe the base eversion path up to the point where the base enters the active site, and thus was used as the reaction coordinate for our PMF calculations in this work.

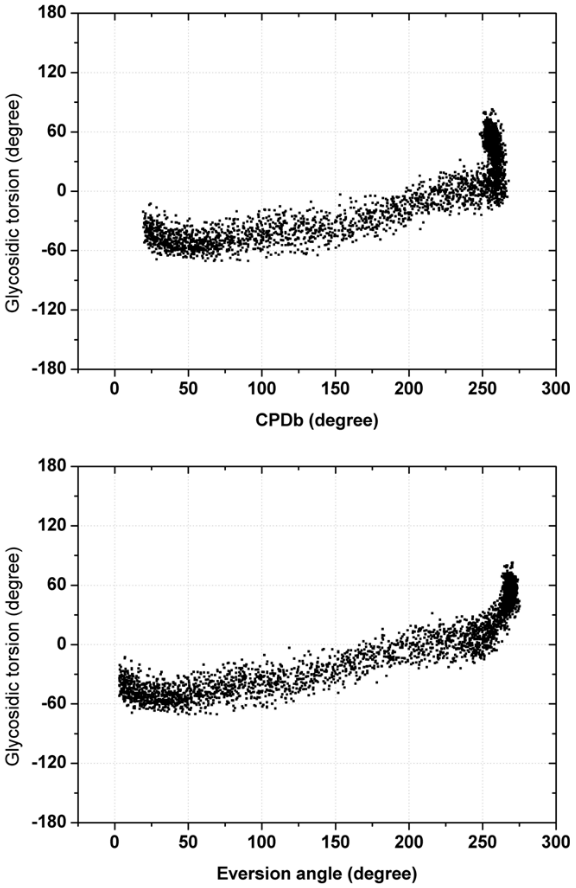


**Figure S1**. Glycosidic torsion angle as a function of CPDb (top) and eversion angle (bottom), showing these two reaction coordinates' sensitivity to the rotation of the glycosidic bond and whether dynamics along the NEB pathway can be adequately described by a single reaction coordinate. Data points are calculated from the NEB production trajectories of the oxoG system. The eversion angle (lower) shows greater ability to discriminate changes in the final part of eversion and thus was chosen as the reaction coordinate used here for umbrella sampling.

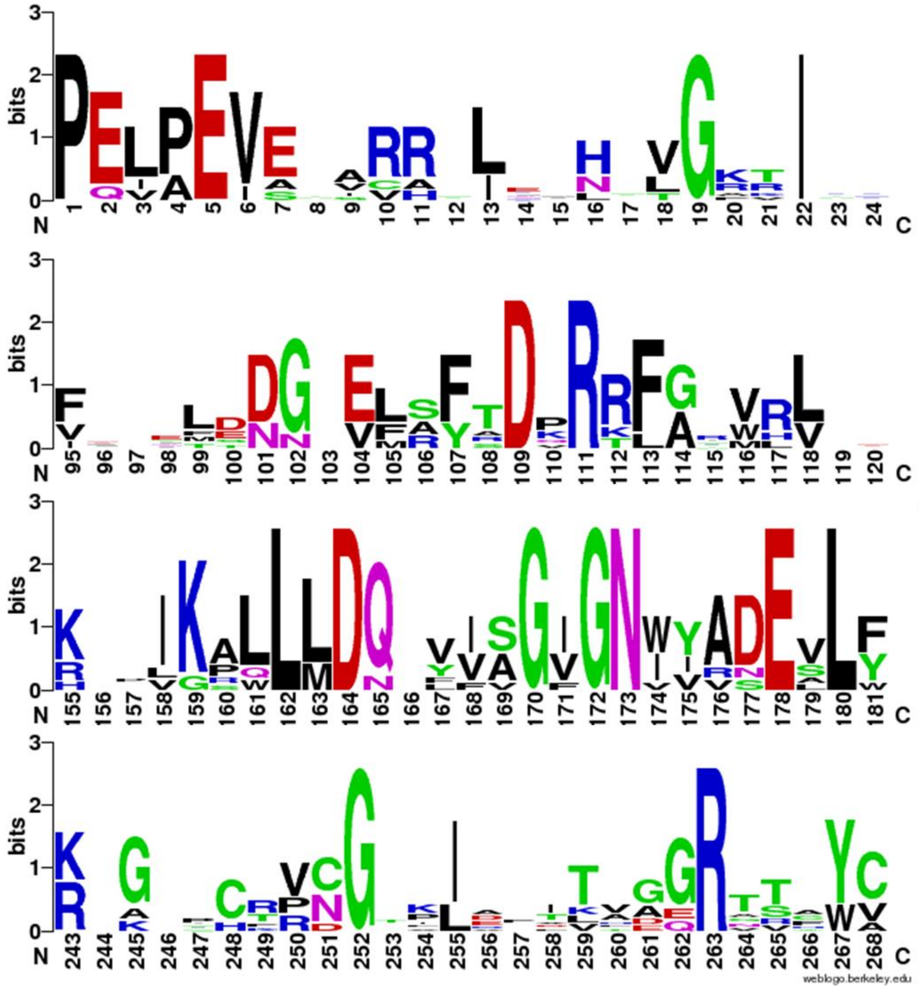


**Figure S2**. Sequence conservation surrounding Asn173 among *G. stearothermophilus* Fpg (gi|38492995), *E. coli* Fpg (gi|15804176), *Mycobacterium tuberculosis* Fpg (gi|148662769), *Arabidopsis thalania* Fpg (gi|18404050), *Oryza sativa* Fpg (gi|115469160), *Candida albicans* Fpg (gi|3850130), and *Neurospora crassa* Fpg (gi|157072070).

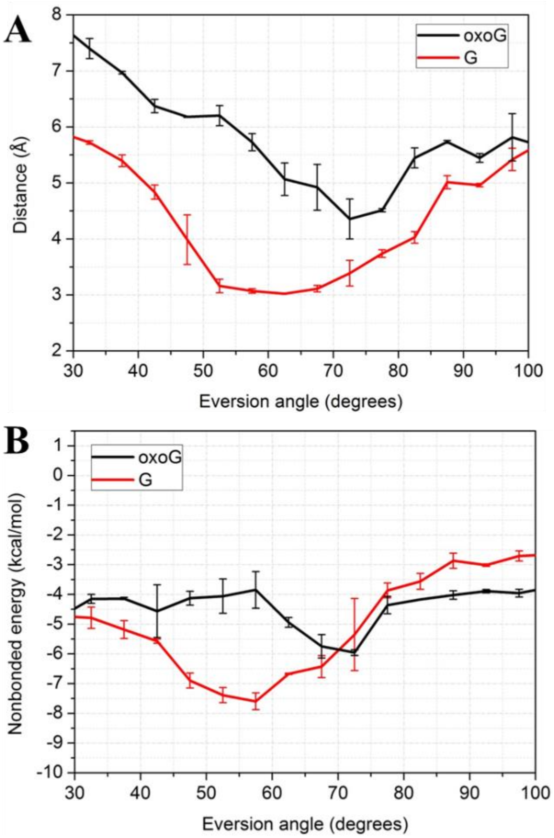


**Figure S3.** A) Comparison of distances between the backbone N atom of Gly264 and the N7 atom of oxoG/G (black/red). The G-Gly264 hydrogen bond forms at an eversion angle of 50°–75°, while oxoG is further away from Gly264. B) Comparison of pairwise nonbonded energies between the base moiety of oxoG/G (black/red) and Arg263. The error bars reflect the difference between two independent US runs.

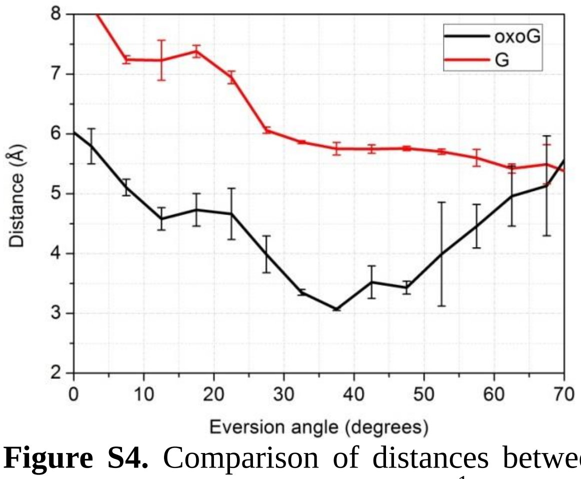


**Figure S4.** Comparison of distances between the O2P atom of $p^1$ and the N7 atom of oxoG (black) and G (red). The oxoG- $p^1$ hydrogen bond was formed at the eversion angle of ~40° (Stage I). The error bars reflect the difference between two independent US runs.

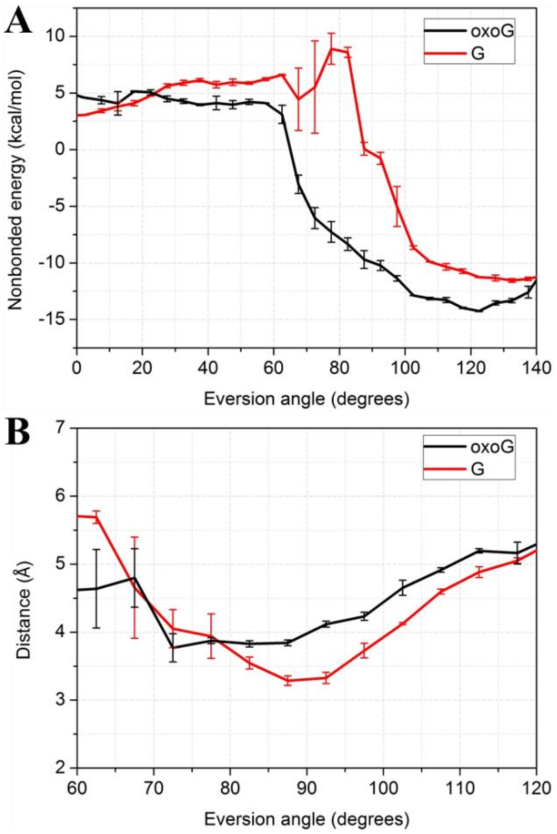


**Figure S5**. A) Comparison of pairwise nonbonded energies between the base moiety of oxoG/G (black/red) and p[1]. B) Comparison of distances between the O6 atom of oxoG/G (black/red) and the Nζ of Lys257. The error bars reflect the difference between two independent US runs.

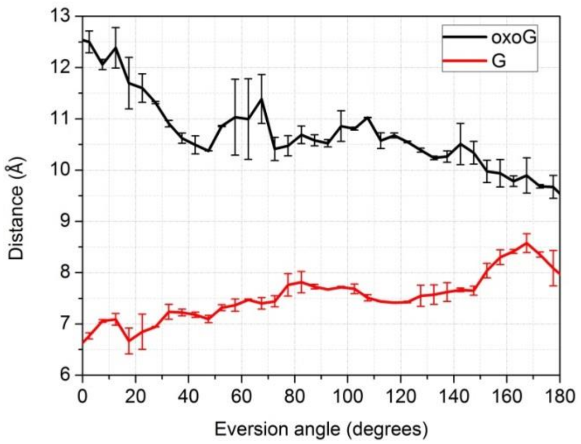


**Figure S6**. Comparison of distances between the Cα of Gly264 and the C3' of the 5' nucleotide of the oxoG (black) and G (red), showing that the gap between the zinc finger hairpin and DNA is wider in for oxoG than for G. The error bars reflect the difference between two independent US runs.

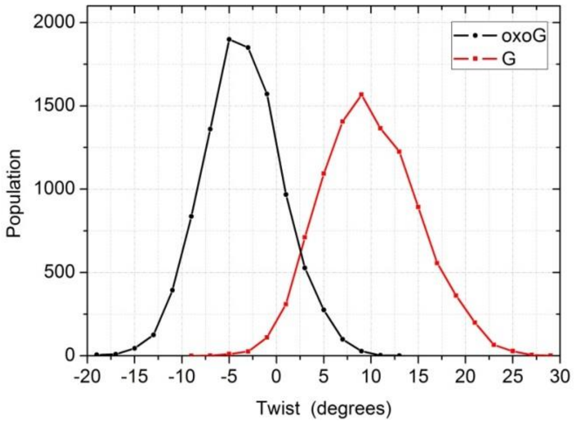


**Figure S7.** Comparison of twist angles between the target and the 5’ base step in the oxoG system (black) and the G system (red). Only the intrahelical windows (the first two windows in each umbrella sampling run) were included in the calculation.

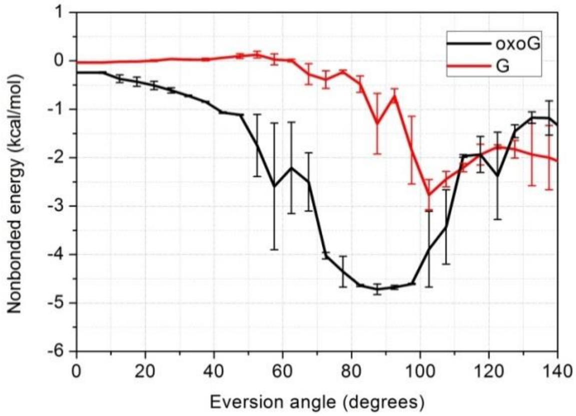


**Figure S8**. Comparison of pairwise nonbonded energies between Asn173 and the base moiety of oxoG (black) and G (red). The error bars reflect the difference between two independent US runs.

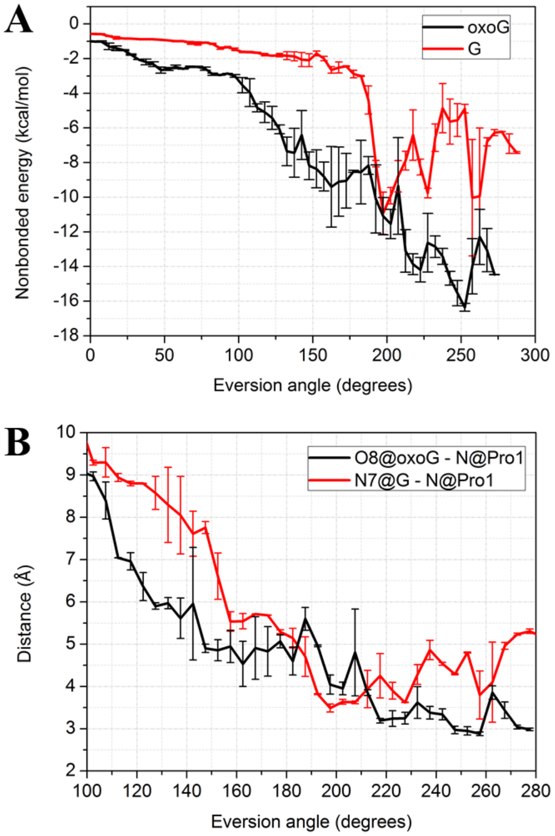


**Figure S9**. A) Comparison of pairwise nonbonded energies between the base moiety of oxoG/G (black/red) and Pro1. B) Comparison of distances from the amine of Pro1 to the $O^8$ of oxoG (black) and to the N7 of G (red). The error bars reflect the difference between two independent US runs.

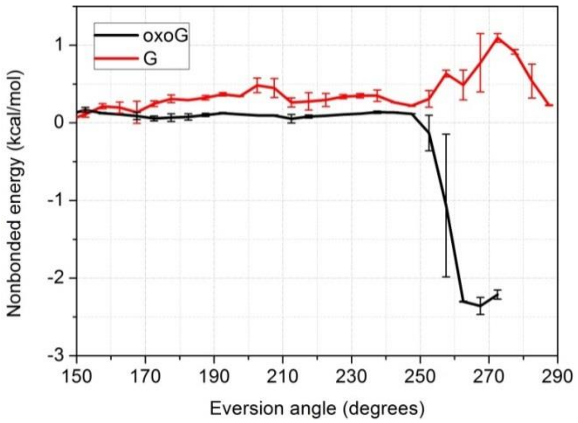


**Figure S10.** Comparison of pairwise nonbonded energies between the base moiety of oxoG/G (black/red) and Ser219 in OCL. The error bars reflect the difference between two independent US runs.

References

1. Song, K.; Kelso, C.; de los Santos, C.; Grollman, A. P.; Simmerling, C., Molecular Simulations reveal a common binding mode for glycosylase binding of oxidatively damaged DNA lesions. *J Am Chem Soc* **2007,** *129* (47), 14536-14537.
